# The Prevalence of Methamphetamine Dependence among Iranian Women in Methadone Maintenance Therapy in Tehran, Iran 

**Published:** 2018-01

**Authors:** Omid Massah, Afsaneh Moradi

**Affiliations:** 1 Substance Abuse and Dependence Research Center, University of Social Welfare and Rehabilitation Sciences, Tehran, Iran.; 2 Department of Psychology and Educational Sciences, Al-Zahra University, Tehran, Iran.

**Keywords:** *Methamphetamine*, *Methadone*, *Prevalence*, *Treatment*, *Women*

## Abstract

**Objective:** To date, there has been no specific survey of the prevalence of methamphetamine (MA) dependence among Iranian women in methadone maintenance therapy (MMT). The current study aimed at addressing this critical gap in the literature.

**Method** **:** This study was part of a larger cross-sectional survey, which was conducted in 36 MMT clinics in Tehran during 2015 and 2016. A researcher-designed questionnaire was devised to collect data on demographics and drug and treatment characteristics. Data were analyzed using SPSS Version 19.

**Results:** Overall, 307 women were in the study sites. Of them, 275 were MA dependent (89.5%) while in MMT. The mean age of the participants, who were MA-dependent, was 38 years. Age of MA dependence was 30 years, and duration of MA dependence was 7years. However, only 24.3% of the participants were currently in MA treatment (i.e. Matrix Model). This was accompanied with high rates of psychiatric hospitalization (29.4%), anxiety (33.4%), and depression (50.9%) because of long years of untreated MA dependence.

**Conclusion: **The study revealed a high prevalence of untreated MA dependence and its adverse health impacts among the participants. However, no considerable treatment for MA dependence had been received by the participants. Psychosocial treatments should address MA dependence in MMT in Iran.

Methamphetamine (MA) is a psychostimulant drug with highly addictive characteristics (1, 2). In recent years, MA dependence has become a health concern in Iran (3, 4). The prevalence of MA dependence is less than 1% in the general population (3). However, MA dependence soars among men and women in methadone maintenance therapy (MMT) (1). The reason associated with this problem includes self-treatment for depression and sexual dysfunction (5-8). 

Has also been specifically reported among Iranian women in MMT (3). This issue is associated with multiple harms such as poor psychological well-being, social dysfunction, and poor motivation to change (1-8).

Studies in Iran indicate that women in MMT are more likely to be dependent on MA because of methadone-related depression and life pressures, while men in MMT were more likely to be dependent on MA because of sexual problems (6-8). 

A study indicated that the lifetime prevalence of MA use was associated with gender, poor knowledge of MA use, and extramarital sex (5).

Studies show that there is no pharmacological treatment for MA dependence (9). The treatment of MA dependence is largely based on psychological treatments, such as the Matrix Model and cognitive-behavioral therapy (10, 11). 

MA dependence in MMT may significantly reduce positive treatment outcomes among women. However, there are few studies of this group of MA-dependent patients in Iran. The present study aimed at determining the prevalence of MA dependence among a group of women in MMT.

## Materials and Methods

*Study Design and Settings*

The study was part of a larger cross-sectional survey of MA dependence in MMT services in Tehran, Iran, which was conducted during 2015 and 2016. Overall, 36 methadone treatment services were selected for the study. The study sites were located in different parts of Tehran, and 5 study sites were women-only. The criterion for selecting the study sites was the number of reported female methadone patients. Half of the study sites were located in middle class areas of Tehran.

*Participants*

MA dependence was defined as meeting the Diagnostic and Statistical Manual of Mental Disorders, fourth edition, third revision (DSM.IV-TR) criteria for MA dependence (12). Other inclusion criteria included age of at least 18 years, female gender, and being in methadone treatment for at least 3months. Exclusion criteria included reporting drug intoxication or withdrawal symptoms at the time of interviewing, which were likely to impact on interview procedures. Half of the participants were recruited from women-only centers.

*Study Measures*

A researcher-made questionnaire was designed to collect the demographic and drug and treatment characteristics of the participants. This included age, education status, living conditions, job status, marriage, lifetime use of illicit drugs, details of MA dependence, duration of methadone treatment, lifetime and present MA treatment, as well as the details of lifetime psychiatric hospitalization and current psychiatric diagnosis. The questionnaire was assessed in a 2-week test-retest on 30 women, and the reliability was found to be 92%, indicating that the reliability of the questionnaire was high.

*Ethical Considerations*

First, participants were informed that the study procedure was voluntary and confidential. Participants were informed that lack of participation would not impact on their MMT. Participants received small gifts for study participation. The study was part of a larger research in Tehran, which was approved by Tehran University. Consent form was obtained from each participant. 

*Statistical Analyses*

Data were analyzed using SPSS Version 19. Descriptive analyses including mean, percent, and standard deviation were used to report demographic, drug, and treatment characteristics of the participants.

*Study Procedures*

The whole study procedures were conducted at the study sites. Participants were interviewed between January 16, 2015 and December 21, 2016. A private interview room was allocated to the study at each centre. Each participant was individually interviewed by a female clinical psychologist.

## Results

Overall, 307 women were in the study sites. Of them, 275 were MA dependent in MMT. This group of MA-dependent women was studied (Table 1). The mean age of the participants was 38 (SD 9.8) years. The majority of the participants (72.7%) had less than nine years of formal education. Most of the participants were living without their families (65.4%), were unemployed (72%), and were currently unmarried (54.5%), meaning that they were single, divorced, or widowed (Table 1).

Participants reported that they initiated drug use with opium (37.5%), heroin (36%), and MA (26.5%), respectively. All the 275 participants were MA dependent on methadone program, meaning that the prevalence of MA dependence was 89.5% among 307 women who were on treatment. The age of the first MA use was 28 (SD 9.1) years, the age of MA dependence was 30 (9.8) years, and duration of MA dependence was 7 (SD 7.6) years (Table 1). 

Some participants reported lifetime MA treatment, which included the Matrix Model (31.6%) and therapeutic community program (40.7%), respectively. Only 24.3% of the participants were currently in MA treatment (i.e. Matrix Model). A considerable number of participants (29.4%) reported lifetime psychiatric hospitalization due to MA dependence. A considerable number of participants reported current psychiatric diagnoses of depression (50.9%) and anxiety (33.4%) because of MA dependence (Table 1).

## Discussion

The current brief report addressed investigating a serious health concern in Iran, which has been underreported (13). Women were almost middle-aged, typically without families, and unemployed. This was consistent with a study in Iran, which indicated that most women in MMT were jobless and lived without their families (14).

All participants had lifetime experience with poly drug use. Furthermore, they were all MA-dependent. The high prevalence of MA dependence among these women in MMT necessitates an immediate treatment. However, a few women were currently in MA treatment, and this is consistent with a study which demonstrated that few women in MMT received MA treatment (13).

A considerable number of participants reported lifetime psychiatric hospitalization due to MA dependence. A considerable number of participants reported current diagnoses of depression and anxiety because of MA dependence. However, no treatment was reported. Psychiatric comorbidity can reduce positive methadone treatment outcomes (8). Therefore, mental health evaluation at psychiatric services should be provided for this group of women. Further studies are suggested to investigate this issue.

As MA dependence continues among Iranian women in MMT, it is necessary to ensure that adequate resources are allocated to treatment. The Matrix Model can be used for the treatment of MA dependence (10, 11) although it may be intensive and long (15). However, the coverage of the treatment or high cost may not be appropriate for all women in MMT. Thus, MMT services should provide cost-effective and short psychological treatments for these women. Cognitive-behavioral therapy in the short-term format may be needed for this group of women in methadone clinics. Further studies are suggested in gender differences and treatment outcomes as indicated in previous research studies (16-18).

**Table 1:** Participant Characteristics (n=275)

**Characteristics**	**Mean/Percent**
Participants	
Age (range 18-65 years)	38 (SD 9.8)
Education status	
Less than nine years	200 (72.7%)
More than nine years	75 (27.3%)
Living conditions	
Without family	180 (65.4%)
With family	95 (34.5%)
Job status	
Unemployed	198 (72%)
Employed	77 (28%)
Marriage	
Currently unmarried	150 (54.5%)
Currently married	125 (45.6%)
First illicit drug of use (lifetime)	
Opium	103 (37.5%)
Heroin	99 (36%)
MA	73 (26.5%)
Current MA dependence	275 (100%)
Age of first MA use	28 (SD 9.1)
Age of MA dependence	30 (SD 9.8)
Duration of MA dependence	7 (SD 7.6)
Route of MA administration	
Smoking	228 (83.0 %)
Injection	24 (8.7%)
Ingestion	23 (8.3%)
Duration of methadone treatment (range 3-49 months)	12 (SD 12.3)
Lifetime MA treatment	
Matrix Model	87 (31.6%)
Therapeutic community treatment	112 (40.7%)
Present MA treatment	67 (24.3%)
Lifetime psychiatric hospitalization*	81 (29.4%)
Current psychiatric diagnosis*	
Depression	140 (50.9%)
Anxiety	92 (33.4%)

* Related to MA dependence

## Limitation

The study has several limitations. First, the study was limited to a group of women in MMT. Therefore, the study findings may not be generalizable to men on methadone program. Second, the study was limited to Tehran. Therefore, the study findings may not be generalizable to other parts of Iran. However, the present research was among few studies that investigated the prevalence of MA dependence among women on methadone program. Future studies should consider the prevalence of MA dependence and gender differences among both men and women on methadone program.

## Conclusion

The study indicated the high prevalence of MA dependence and its adverse health impacts among the participants. However, no considerable treatment for MA dependence had been received by the participants in MMT. Although some women had received the Matrix model and cognitive-behavioral therapy, the treatment coverage was limited. Psychosocial treatments should address MA dependence and its health impacts on women in MMT on a large scale in Iran.
